# Urological Tract Infection with Candida Bezoars in a Woman with Diabetes Type 2

**DOI:** 10.1155/2021/5563030

**Published:** 2021-03-08

**Authors:** Astrid Van Daele, Johan Schurmans, Raf Van Reusel

**Affiliations:** AZ Turnhout, Rubensstraat 166, 2300 Turnhout, Belgium

## Abstract

The aim of this report is to describe a case of fungal bezoar obstruction of the kidney and our experience in managing it endoscopically. A 61-year-old woman presented with flank pain. On CT scan, a proximal stone and secondary dilatation was seen. After placing a DJ stent and administering antibiotics, there was persistent pain, sickness, and kidney dilatation on CT scan. URS showed soft tissue bezoars surrounding the stent and the stone. We managed to remove all the fungal balls mechanically and endoscopically. This report showed that endoscopic removal of the bezoars is a good alternative for antifungal installation.

## 1. Introduction

Urinary tract infections caused by fungi are not rare. The most common species is Candida albicans, which is responsible for over 50% of the cases [1].

The organism exists as a commensal and is most frequently found on the skin. In immunocompromised or debilitated individuals, the fungus may become a pathogen. Hosts with certain risk factors can be susceptible to this opportunistic infection. Urinary tract colonization can be the result of a systemic, disseminated infection or of a local, retrograde infection of the bladder.

Known risk factors include recent or long antibiotic use, diabetes mellitus, indwelling catheter use, urinary tract pathology, malignancy, pregnancy, immunosuppression, renal transplantation, and recent surgery. Also urinary stasis and lithiasis have been reported [2].

Rarely, the fungus can be organized in fungus balls or bezoars, colonizing the drainage system, sometimes leading to obstruction.

## 2. Case Presentation

A 61-year-old woman, known with diabetes type 2, presented at the emergency department with left flank pain and no fever. White blood cell count (WBC) was 10 000/*μ*L, C-reactive protein (CRP) 32 mg/dL, and glomerular filtration rate (GFR) 58. No imaging was done, and the patient was sent home with Levofloxacin and the diagnosis of an ascending urinary infection.

Two days later, the patient came back to the ER with persisting pain and still no fever. Now the white blood cell count was 11 000/*μ*L and C-reactive protein 8 mg/dL. A CT scan without contrast was done and showed a proximal stone of 6 mm in the left ureter and a secondary dilated pelvicalyceal system. No other aberrant findings were described in the urinary tract. Urinary culture from two days ago showed E. coli, sensitive to the taken antibiotics.

The patient got admitted to the urology department, and a DJ stent was placed. She was discharged on her own initiative the day after with continuation of the antibiotics.

**Figure 1:**
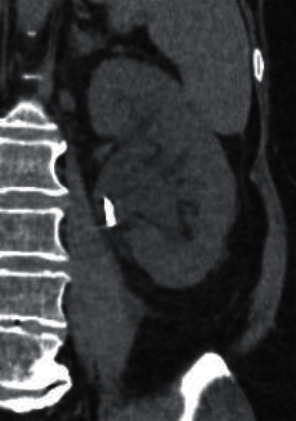
CT scan; persistent hydronephrosis despite DJ stent.

Three days after discharge we saw her back at the consultation, earlier than planned. She felt sick, was vomiting, and reported persistent flank pain without fever. Repeat blood sample, urine sample, and blood cultures were taken, and she was admitted. Her WBC was 9000, CRP 200 and GFR 41. A repeat CT scan showed persisting dilation of the left kidney, DJ stent, and stone unchanged in situ (Figure 1).

We decided to perform a diagnostic cystoureteroscopy, thinking the stent was maybe dysfunctional or not well positioned.

**Figure 2:**
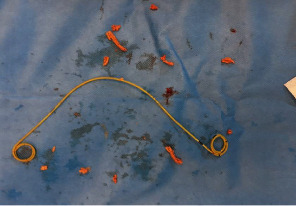
DJ stent and surrounding fungal tissue.

On cystoscopy and ureteroscopy, we found soft, spongy masses in the bladder and along the stent (Figure 2). We removed the tissue from the bladder mechanically with small forceps and by rinsing it out through the cystoscope.

In the kidney, we aspirated the mass and pus with syringes through the ureteroscope. We sent this together with the bezoars for culture and microscopy. We removed the stone and the stent and placed no bladder catheter.

Candida albicans was then isolated from the urine, the aspirated fluid and tissue, and her blood. In addition, we isolated E. coli from the urine.

After consulting a microbiologist, we put her on intravenous Diflucan 400 mg/day and Levofloxacin 500 mg twice a day. Two days later, we additionally started her on oral and vaginal antifungal agent because of local Candida infection. Blood cultures were carried out daily. We continued the Diflucan until 14 days after the first negative blood culture.

Fundoscopy and transesophageal ultrasound were carried out to exclude intraocular infection and endocarditis. They were negative.

On outpatient consultation, she did well and blood and urine cultures were negative.

## 3. Discussion

In our case, the patient was only known with diabetes as the predisposing condition. The trigger for the infection was the proximal ureter stone. On the third admission, her glycaemia was too high. This could be due to the infection she was going through. On the other hand, the uncontrolled glycaemia could have made her susceptible for the Candida infection.

In all debilitated or immunosuppressed (septic) patients who have upper urinary tract obstruction with renal filling defect, fungal infection should be considered.

The clinical diagnosis can be confirmed by positive urine or hemoculture. The presence of obstruction and inflammation without lithiasis or after placement of a stent, in the renal collecting system, could be an important clue in the radiological diagnosis. On the ultrasound, an enlarged kidney filled with fungus balls can be seen. On CT, the fungus balls appear as soft tissue masses in the collecting system and, depending on the volume, with mass effect. On contrast-enhanced CT, the tendency of obstruction can be objectivized [3].

Fisher et al. published an algorithm for the treatment of Candida urinary tract infections in general. For bezoars and mycetomas, it is stated that, next to systemic therapy with antifungal agents (fluconazole is preferred), surgical drainage is almost always needed [4].

In the case of presence of fungal balls, several methods have been proposed, such as installation through nephrostomy and percutaneous endoscopic disruption of percutaneous irrigation with streptokinase.

In our case, we managed to physically remove all masses endoscopically, so there was no need for antifungal installation. As Abuelnaga et al. also demonstrated, endoscopic retrieval of the renal tissues by ureteroscopy can be an effective and rapid way to manage fungal balls. The hospital stay and toxicity associated with antifungals can hereby be reduced, in comparison to installation through nephrostomy [5]. Especially in cases where a diagnostic URS is carried out or in cases where a nephrostomy is contraindicated, the URS should be considered as a first line treatment, in combination with systemic antifungal treatment.

## 4. Conclusion

We described a case of a patient, predisposed with diabetes type 2, who suffered renal obstruction due to a Candida infection with fungal bezoars.

In this case, we managed to physically remove all masses endoscopically, so there was no need for antifungal installation. Especially in cases where a diagnostic URS is carried out or in cases where a nephrostomy is contraindicated, the URS should be considered as a first line treatment, in combination with systemic antifungal treatment.
